# Experiences and opinions of multi-professional non-medical oncology prescribers on post-qualification training: a qualitative study

**DOI:** 10.1007/s11096-022-01396-6

**Published:** 2022-04-05

**Authors:** Sophie E. Harding, Christopher A. Langley, Annabel Borley, Bethan Tranter, David R. P. Terry

**Affiliations:** 1 Velindre Cancer Centre, Cardiff, UK; 2 Aston Pharmacy School, Aston University, Birmingham, UK

**Keywords:** Drug prescriptions, Education, Medical oncology, Nurses, Pharmacists, Radiology

## Abstract

*Background*: Within the UK, a non-medical prescriber is a non-medical healthcare professional who has undertaken post-registration training to gain prescribing rights. Lack of post-qualification NMP training has previously been identified as a barrier to the development of oncology non-medical prescribing practice. *Aim*: To explore the experiences and opinions of multi-professional non-medical oncology prescribers on post-qualification training. *Method*: Nine out of 30 oncology non-medical prescribers (three nurses, three pharmacists and three radiographers) from a single cancer centre in Wales, were selected from a study site NMP database using randomisation sampling within Microsoft® Excel. Participants were interviewed using a validated and piloted semi-structured interview design on the topic of post-qualification training for non-medical prescribers. Participants were invited via organisational email. Interviews were audio-recorded and transcribed verbatim. Anonymised data were thematically analysed aided by NVivo® software. *Results*: Main themes identified: experience related to training, competency, support and training methods. Competency assessment methods discussed were the annual non-medical prescriber appraisal, peer review and a line manager’s overarching appraisal. Support requirements identified included greater consultant input to help non-medical prescribers identify training and peer support opportunities. Organisational support was requested regarding regular study leave and governance around clinical judgement and errors. The need for regular structured in-house training related to non-medical prescriber’s level of experience was identified. *Conclusion*: Development of organisation-led governance strategies and in-house training programmes will support training equity for all non-medical prescribers within the organisation.

## Impact on practice

- Non-medical prescribing competence incorporating a variety of assessment methods and tools, such as OSCEs, peer review and ‘portfolios of evidence’, will support maintenance of non-medical competence.
- Implementation of an organisation-wide oncology non-medical prescribing training programme, based on level of experience, will guide non-medical prescribers and their consultant mentors with support and aid transition from early career to advanced practice.
- Establishing consultant roles for each non-medical prescribing profession will provide leadership and development of non-medical prescribing governance strategies, to support training equity for all non-medical prescribers.

## Introduction

Within the United Kingdom, a non-medical prescriber (NMP) is a non-medical healthcare professional, who has undertaken post-registration university training to gain prescribing rights [1].

NMPs are either an ‘independent’ or ‘supplementary’ prescriber. An independent prescriber (IP) is defined as ‘a practitioner, who is responsible and accountable for the assessment and diagnosis of patients’ conditions and can make prescribing decisions to manage the clinical condition of the patient’. Professionals who can qualify as IPs in the UK are midwives, nurses, optometrists, paramedics, pharmacists, physiotherapists, podiatrists, and therapeutic radiographers [2]. A supplementary prescriber (SP) is a practitioner who can prescribe within the scope of a pre-agreed Clinical Management Plan (CMP) which was implemented and agreed between an independent prescriber (doctor/dentist), an SP and the patient, to manage the patient’s condition. In the UK to date, diagnostic radiographers and dietitians are able to register as SPs only [2].

The main objectives of implementing non-medical prescribing were to improve access to medicines, utilise eligible professionals’ clinical skills and to reduce the workloads of medical colleagues so they could focus on more complicated patient cases [3]. NMPs have become integral to the UK’s National Health Service (NHS) when delivering patient services. Although an NMP workforce is developing at pace in the UK, where the education and training provision of healthcare staff is highly regulated and supported, there is a definite gap regarding NMP post-qualifying training as there are no training specific national competency frameworks or training guidance inclusive of all multidisciplinary NMPs once qualified. This issue was identified within a recent scoping review [4] and an e-Delphi survey establishing priorities in Wales [5]. Unstructured continuing professional development (CPD) opportunities may arise across the country dependant on the sector and specialism of healthcare in which the individual NMP practises. The need for each UK organisation to develop a sound infrastructure and governance pathways for NMP CPD to progress safely within their practice was highlighted within a national study of nurse independent prescribing [6].

Within oncology, all treatments given to treat cancer are known as systemic anti-cancer therapy (SACT) [7]. The NMP workforce contribute significantly to SACT service delivery. Once qualified, oncology NMPs work alongside oncology or haematology consultants prescribing SACT and supportive treatments for cancer therapy in a variety of roles [8]. The oncology NMP role benefits medical prescribers by easing some of the burden of routine prescribing/patient care and ensuring cancer services are responsive to patients’ needs at a difficult time in their lives [8]. There is limited published literature of NMPs within oncology except a national survey exploring specialist nurse prescribing within cancer care [9] and another nurse-led study around prescribing SACT in an oncology outpatient setting [10]. A further study explored the impact of pharmacy services collaboration on improving adverse drugs reactions in cancer chemotherapy [11]. There were no published studies concerning training requirements of oncology NMPs post qualifying, although a NMP guideline has been produced by the British Oncology Pharmacy Association (BOPA) which includes a competency framework for pharmacist NMPs [8].

In 2018, the study site had 30 non-medical prescribers: eight pharmacists, 18 nurses (including two nurse supplementary prescribers (SPs)) and four therapeutic radiographers. Nurse SPs were excluded from this study as their practise varied due to clinical restrictions within CMPs which may affect their training requirements.

At the study site, the majority of nurse and pharmacist NMPs practise within medical consultant oncologist teams reviewing outpatients prior to their next cycle of SACT. NMPs prescribe SACT and supportive medicines, assess haematological tests and request scans and blood transfusions if appropriate. Three nurse NMPs review patients at ward level. Therapeutic radiographer NMPs review patients within radiotherapy review clinics, prescribing supportive medicines and SACT in combination with radiotherapy [12].

NMPs have varying levels of experience at the study site. For this study, NMPs were classified as an ‘advanced practice’ NMP if they have practised within oncology as an NMP for five years or more; if the NMP has practised within oncology for less than five years, they were classified as an ‘early years’ NMP.

Currently, there is no structured programme for NMP training at the study site, although ad-hoc NMP training sessions on various topics are arranged through quarterly NMP meetings, known as the ‘NMP meeting forum’ if needed and mostly arranged by NMP staff themselves. There is no automatic provision of study leave for NMPs, although some NMPs will undertake certain CPD training dependant on their day-to-day role, e.g., Clinical Nurse Specialists (CNS) have an annual CPD training day. All NHS employees at the study site have an annual appraisal with their departmental line manager. All practising NMPs also have an additional annual NMP appraisal with their consultant oncologist mentor as a governance requirement of the organisation. Previous studies undertaken at the study site by Harding and colleagues have explored the opinions and beliefs of all oncology prescribers and their senior managers. They concluded that skills and training of oncology NMPs across the study site organisation needed to be strengthened for NMP practice to develop further.

## Aim

To explore the experiences and opinions of multi-professional non-medical oncology prescribers on post-qualification training.

## Ethics approval

Ethical approval for the study was obtained from Aston University Ethics Committee (Approval ref: 158–2016-SH), on approval date 30/09/2016 (minor amendment in April 2021 adding therapeutic radiographers due to practice change) [13]. Organisational R&D approval was obtained from the study site for the study to proceed but the organisation stated that NHS ethics approval was not required. There was no patient or public involvement in this study.

## Method

A literature search (September 2021) via EMBASE and MEDLINE identified 28 relevant NMP studies published within the last 15 years (since non-medical practitioners were allowed to qualify as NMPs) and focused on non-medical prescribing by pharmacists, nurses and radiographers and narrowed further using education and oncology. MeSH terms were used. Studies were included if they included the study of UK NMP practice and published in English, whilst conference abstracts and publications not published in English were excluded. Although many were predominantly focused on nurse prescribing, only three studies were identified related to oncology NMP practice; none was associated with oncology NMP training [9–11].

### Study site

The research study was set within one oncology centre in Wales, which employs 670 staff from a range of professions across all departments. Each year, 5000 new referrals are received and around 50,000 outpatients are treated. [14].

Between January and June 2018, nine participants were randomly selected to participate in a 60-minute one-to-one face-to-face semi-structured interview (SSI). A randomisation calculation within Microsoft® Excel software (version 15.0) was used to choose potential participants within each professional group. The interview schedule was designed for a 60-minute interview using McNamara guidelines [15]. Interview questions were reviewed using think aloud testing by the researchers. Interviews were piloted by one NMP participant from each professional group who were selected at random (total of three pilot interviews). No changes to interview questions were needed following the pilot. Three participants were randomly selected, irrespective of any other confounders such as length of practice from each of the three NMP professional staff groups practising at the study site [pharmacist group (*n* = 8), nurse group (*n* = 16) and radiographer group (*n* = 4)].Three SSIs from each of the three professional groups were included as this was considered sufficient to explore both the depth and width of the study site, and on analysis findings were considered to be reliable and valid. Further interviews were considered but believed unlikely to add anything of importance. SPs were excluded from the study. Each of the pre-selected nine participants were emailed by the research team using the organisation’s email directory for recruitment and contained both the participant information sheet and a consent form. Provisions were made to recruit more participants using the randomisation software if needed. Participants were asked to sign the written consent form and submit it before the start of the SSI. The study was designed and conducted in accordance with COREQ principles [16].

### Data collection

Face-to-face SSIs were undertaken using reflexivity (by SH) at the study site. The SSIs were audio recorded and included pre-piloted topic questions which were validated by the R&D department at the study site and the pilot interviews were included in the main study data. Free discussion was encouraged around open questions on the topic of NMP training and aimed to explore topics identified within published literature (see Box 1 for interview questions).

### Data analysis

Recordings from all nine interviews were transcribed verbatim by the lead researcher (SH) then anonymised and analysed using a thematic approach (by SH; all transcripts and analysis checked for accuracy by AB) [17], aided by NVivo® software (version 12). Analysis involved coding data and emerging themes were developed and further refined. Representative quotations have been used to evidence and support the analysis, each coded to represent the participant profession. Data protection measures were adhered to for storage and collection of data.

## Results

All participants were IPs practising within oncology at the study site at the time of the interviews. Participants were three nurse NMPs, three pharmacist NMPs and three radiographer NMPs.

Analysis using a thematic approach of the data identified four main themes: experience relating to training, competency, support and training methods. Sub-themes were also identified (see Table 1).

### NMP experience relating to training requirements

Table 2 displays study participant demographics and their number of years’ experience. The post-qualifying NMP training participants had received is also within Table 2 grouped for each professional group. Similarities between the current NMP training experienced by participants are displayed within Table 2. Table 3 shows when the study participants believe the NMP training topics (within Table 2) should be completed by the oncology NMP, categorised by the NMP’s level of experience.

#### Other future training suggested by study participants

Participants from all professional groups recommended that structured in-house training for all NMPs is needed. Table 4 shows a list of future training topic ideas per professional NMP group.

### NMP competency

Respondents discussed several areas relating to competency, NMP appraisal, objective structured clinical examinations (OSCEs), scope of practice and peer review. Exemplar quotes are provided in Box 2.

#### NMP appraisal

Nurse-1, Pharmacist-2 and Radiographer-1 described varying opinions on the use of annual revalidation appraisals as a method of ensuring competency governance at the study site. Nurse-1 discussed undertaking a combined appraisal method which involved NMPs proactively collecting a competency ‘portfolio of evidence’ which was reviewed by their nursing line manager and signed off by the consultant oncologist.

#### OSCEs

An OSCE is a direct observation evaluation tool used to assess clinical staff [18]. Nurse-1 described previously using OSCEs as a revalidation tool for their NMP practice. Nurse-1 believed that assessing NMPs’ clinical reasoning and judgement were more important than completing regular OSCEs for more experienced NMPs.

#### Scope of practice

Nurse-1 suggested that each NMP’s scope is not clearly defined at the same level of detail across all oncology specialities, which may be due to variable expectations of consultant mentors.

#### Peer review

Pharmacist-1 and Pharmacist-3 believed that peer review could be used to assess competency. Nurse-1 recommended developing an oncology ‘independent prescribing advanced practice framework’ which could be used to peer review NMPs by reviewing their portfolios of evidence.

Nurse-2 described how they audited themselves as a method of assessing competency, which could have been undertaken due to lack of peer review available at the study site. Collecting and recording NMP prescribing data is not current practice at the study site.

### Support for NMPs

All participants agreed that training support from all stakeholders is fundamental to the NMP role. Exemplar quotes are provided in Box 3.

#### Consultant support

Most participants stated that they did receive training support from their consultant oncologist, although they also described a need for more leadership and support with their NMP development. Pharmacist-3 explained that they used their own initiative to direct their own learning and training needs.

#### Line manager support

Pharmacist-1 and Pharmacist-2 described a lack of support for attending NMP training from their line manager, due to their line manager not being an NMP themselves, but there was support from the NMP lead. Radiographer-1, Radiographer-2, Radiographer-3 and Nurse-2 described feeling supported by their line managers regarding NMP training. This may be due to their line manager also practising as an NMP. The consultant non-medical professional role was not discussed by pharmacists and nurses due to this role not being implemented within their departments at the study site. Radiographer-3 did describe their consultant radiographer currently providing NMP leadership within the radiotherapy department at the study site.

#### NMP peer support within the organisation

All participants described how future in-house NMP training sessions are planned within the study site NMP meeting forum on an ‘ad hoc’ basis. Pharmacist-3 suggested that these meetings could be improved upon, especially regarding their frequency and efficacy. Nurse-2 suggested utilising the NMP meeting forum to facilitate peer support, i.e., more experienced NMPs sharing experiences with early career NMPs.

#### Organisational support

Nurse-1 and Nurse-2 commented on organisational support. Nurse-1 described the need for support when NMPs make an error, making decisions and for regulation protection regarding their clinical judgement. Nurse-2 believed that regular study leave should be provided by the organisation for NMPs’ training mirroring the current medical prescribers’ training at the study site.

#### Professional group support

Regarding professional support internal to the organisation, Radiographer-1 and Radiographer-2 described having internal radiographer department support sessions for case discussion and in-house NMP competencies led by the lead radiographer NMP (Consultant Radiographer) and believed these to be beneficial. Pharmacist-1, Pharmacist-2, Pharmacist-3 and Nurse-1, Nurse-2 and Nurse-3 did not describe any internal professional training support across the study site, although Nurse-2 described attending useful regular CPD sessions within their CNS role.

Regarding professional support external to the organisation, Pharmacist-1 described utilising British Oncology Pharmacy Association (BOPA) events to keep up to date and network with external colleagues. Radiographer-1 described a radiographer national online forum for NMPs. Both Pharmacist-1 and Radiographer-1 found these beneficial to their practice.

### Methods of providing training

Main training methods described by participants are detailed below with exemplar quotes in Box 4.

#### Learning from others

All participants commented on various aspects of ‘learning from others’. Nurse-1 discussed how their practice benefitted by communicating with other nurses fulfilling the same role, both within and outside of their organisation. Learning from other NMP professional groups was described as beneficial by Nurse-3.

#### In-house training

Nurse-2 explained how they had not completed any formal training after qualifying as an NMP. Nurse-3 believed that in-house training should be available on a regular basis and Radiographer-3 was in favour of regular in-house training, but topics should be beneficial to all. Pharmacist-3 described how NMPs should be allocated time to attend established in-house junior medical training within the organisation. Participants made suggestions for the development of a structured in-house organisation-wide NMP training programme.

#### Self-directed Learning

Seven NMP participants described using their own initiative to undertake relevant training to develop their own NMP role. Nurse-2 had experienced a lack of guidance when extending their scope of practice and believed that more guidance should be available from the organisation.

## Discussion

### Key findings

NMP appraisal was the main method of competency assessment described at the study site and its structure was believed to vary between different NMP professional roles or between NMP individuals. One participant described a line manager NMP appraisal method of assessing competency which could be made available to all NMPs. Another participant believed that an NMP’s scope of practice should be clearly defined; this does not currently occur, although it is requested within the organisation’s NMP guideline. Peer review of a portfolio of evidence was not currently utilised for NMP competency assessment at the study site, but participants believed it would be beneficial to implement.

Most study participants felt supported by their consultant mentor but believed their consultant mentors should have more responsibility for sharing their views on an individual’s NMP training needs. The level of line manager NMP support experienced by participants was believed to be affected by line managers’ understanding of the NMP role. Participants believed organisational support for NMP training should be viewed as high importance if NMP practice is seen as important to the organisation’s strategy. Departmental support sessions were in place for radiographer NMPs and led by a radiographer lead NMP (Consultant Radiographer). However, nurses and pharmacists within the organisation did not describe any designated departmental professional NMP leads.

Various learning methods for NMP training were described by each professional NMP group. Nurse NMPs train and discuss practice with their nursing colleagues within the same role, radiographers learn within departmental peer review sessions, and pharmacist NMPs learn from other pharmacist NMP peers outside of the organisation. No formal training after qualifying as an NMP was in place and developing an in-house rolling training programme incorporating training for both early years NMP practice and advanced level NMPs was recommended to aid oncology NMP development at the study site.

### Strengths and limitations

Prior to this study, oncology NMP training was under-explored within published literature, especially the inclusion of equal representation of pharmacist, nurse and radiographer NMPs. Semi-structured interviews allowed the researcher to explore participants’ opinions in depth.

Trustworthiness in qualitative studies involves ensuring credibility, transferability, dependability and confirmability [19, 20]. The credibility of the study data was supported by randomly selecting participants, but a greater number could have been included to improve credibility further and potentially reach data saturation. Random selection also did not allow selection of participants with a variation in experiences as an NMP as many of the participants included were advanced level NMPs. Dependability and confirmability were ensured by having the analysis checked by another researcher following initial analysis by the lead researcher (SH).

Although participants were all employed and practised at one study site, experiences and opinions on NMP competency and support may vary within other organisations. Therefore, study findings may or may not be transferable to other organisations or other medical specialties. Triangulation was not used within the study methodology but could have established a higher level of trustworthiness of the study data.

### Interpretation

Organisation-wide adoption of the combined NMP and line manager appraisal method model, which involves a review of each individuals’ portfolio of evidence (previously signed off by the consultant mentor) within an annual line manager appraisal, is in line with BOPA recommendations on competency assessment [8]. At the study site, clear details of NMP competencies are recognised within the organisation’s NMP guidelines, but were not linked with appropriate training opportunities [2]. Participants described OSCEs as a revalidation tool but believed they would be utilised more effectively for assessing clinical reasoning and judgement. However, a qualitative study exploring how pharmacists and nurses make clinical decisions by Abuzour and colleagues discussed complexity of assessing NMP clinical reasoning, so further investigation into the suitability of OSCEs to assess clinical reasoning within this group is warranted [21]. Peer review was suggested involving a panel of oncology experts within the organisation reviewing a portfolio of evidence. A national study by Smith and colleagues exploring the safety and quality of nurse IP found that 52% of nurse respondents also used peer review to review their practice but did not provide detail on how and by whom [6]. Prescribing audits could also be used organisation-wide to feedback to NMPs and would demonstrate prescribing patterns. A cross-sectional survey of newly registered pharmacist views by McIntosh and colleagues and national study by Smith and colleagues reported that pharmacists and nurses both either identified prescribing auditing as a training need or as already utilised as a quality assurance tool within their organisations [6, 22]. Study findings around competency assessment support the recommendation by BOPA to use a combination of methods [8]. A study exploring the CPD needs of nursing and allied healthcare professionals stated that the employer is ultimately responsible for ensuring prescribing competency [23, 24].

Consultant support is seen as beneficial to NMPs, the study by Latter and colleagues also described how the benefit of medical support can build NMP confidence [25]. Varying support from line managers may have been affected by variability in departmental training budgets and staffing levels allowing them to enable access to NMP CPD courses. Further improvement in NMP peer support is required and recommended using a ‘forum approach’ where more experienced NMPs share their expertise with early career NMPs. A ‘buddy’ mentoring system has been previously recommended to provide extra support for early career NMPs in two studies, exploring the use of a mentoring scheme for NMPs and factors affecting willingness to take responsibility for NMP [26, 27]. The Department of Health (DoH) and the e-Delphi survey study describes how each organisation should have NMP outline strategies for NMP development including providing adequate support for NMPs [5, 28]. Pharmacist NMPs may not have opportunities to share their NMP experiences and knowledge with pharmacist peers and establishing professional peer groups has been recommended to aid development of pharmacist prescribers within a scoping review of barriers to pharmacist prescribing [4]. Creating a specific consultant pharmacist role could also lead on implementing a pharmacist-specific NMP development pathway within the organisation, including setting up a pharmacist NMP peer discussion forum as suggested within one strategic health authority study [29].

A need for a post-qualifying training programme related to competency was highlighted by study participants and the development of formal programmes of CPD have been recommended as a priority for all employers in two nursing studies exploring nurse prescriber CPD [23, 30]. A UK study exploring stakeholder views of nurse and pharmacist SP discussed how some pharmacist NMPs considered inter-professional training courses as advantageous, whilst others did not [31]. Picton and colleagues, whilst exploring the need for a prescribing competency framework to address polypharmacy, discussed significant benefits to multi-professional training [32]. The need for a multi-professional oncology NMP training framework is further strengthened due to a projected increase in registered pharmacists becoming IPs by 2025 [33], and because all UK newly qualified pharmacists will be registered IPs from 2026 [34]. Structured NMP training for registered NMPs will therefore enable organisations to attract and retain staff in the future.

### Future research

Themes identified could be further explored in more detail within focus groups, including representation from NMP stakeholders such as current NMP staff, medical staff, and hospital managers both across the organisation and within other organisations offering oncology services. Survey methods could also be used to obtain more generalisable data across a wider participant population and include greater participant numbers. Future development of an underpinning research framework would explore reasons for the lack of training implementation in secondary care settings.

### Recommendations for practice

The combined NMP and line manager appraisal method model suggested should be adopted across the organisation incorporating the portfolio of evidence (which is in line with BOPA recommendations) [8].

Establishing consultant posts for all NMP professional groups within the organisation may provide equitable and strengthened support to all professional NMP groups. These lead professionals could deliver a clinical educator role for oncology NMPs within their departments, but also form an NMP leadership panel. The leadership panel would lead on implementation of a NMP training programme for all professionals, as well as development of their own professional group. The panel could hold a central NMP training budget and manage training allocations and lead on the development of clear organisational NMP governance strategies. The panel would provide support and mentorship to address current NMP training inequalities identified by participants.

Development of a core multidisciplinary NMP programme with separate training on certain topics for certain professional groups is recommended. Future training topics suggested in this study should be considered and incorporated into a training framework algorithm for oncology NMPs within the study organisation with consideration of the NMPs’ level of experience.

## Conclusion

This study explored experiences and opinions of oncology NMPs of current post-qualification NMP training across three professional groups. Lack of a multi-disciplinary standardised approach to NMP post-qualifying training needs to be addressed by the organisation. An organisation-led NMP training programme should be developed which incorporates NMP levels of experience and professional needs. The programme should be supported by a sound organisation-wide NMP training strategy incorporating factors such as support networks and competency assessment, in order to aid development and bolster resilience of future oncology NMP practice.

**Box 1:** Study interview questions

• Describe your general experiences of NMP skills and training post university qualification?
• How do you think your individual skills complement the clinic?
• What is your opinion on the skills and training that should be available to NMPs?
• How can the changes suggested be implemented?
• Are there any courses that you feel you would like to attend or have attended and what are the hindrances of having attended/attending?
• What support do you feel should be available to improve skills and training?

**Table 1:** List of themes and sub-themes identified from SSI transcript analysis

Main themes	Sub-themes
1. NMP competency	NMP appraisals
	Objective structured clinical examinations (OSCEs)
	Scope of practice
	Peer review
2. Support for NMPs	Consultant support
	Line manager support
	NMP peer support within the organisation
	Organisational support
	Professional group support
3. NMP experience relating to training requirements	Early years training
	Advanced practice training
	Other future training suggestions
4. Methods of providing training	Learning from others
	In-house training
	Self-directed learning

**Table 2:** Study participants per professional group and their collective training completed post-qualifying as identified within their semi-structured interviews

Professional Group	Years qualified as NMP	No. of participants from each professional group	Post-qualifying training completed by participant professional group
Nurse	Range (8 years to 14 years)	3	Advanced communication course
			Blood transfusion course
			Clinical assessment course
			Introduction to prescribing
			IRMER^a^ course
			Microbiology course
			Oncology site specific training
Pharmacist	Range (8 years to 14 years)	3	Blood transfusion course
			Clinical assessment course
			Introduction to prescribing
			IRMER^a^ course
			Oncology site specific training
Radiographer	Range (3 years to 10^b^ years)	3	Advanced communication course
			Clinical assessment course
			Financial support course
			Psychology course
			Oncology site specific training
Total		9	

^a^IRMER – Ionising radiation (medical exposure) regulations

^b^Years practising as an NMP, but not as an independent prescriber due to regulations allowing radiographer independent prescribing only being introduced in 2016

**Table 3:** The training courses identified to be relevant to NMP training development related to the level of oncology NMP experience

Early years NMP (Up to five years’ practice as an NMP)	Advanced Practice NMP (More than five years’ practice as an NMP)
Clinical assessment course	Advanced communication course
Financial support course	Blood transfusion course
Introduction to prescribing	Microbiology course
IRMER^a^ course	Oncology site specific training e.g. new treatments
Psychology course	

^a^IRMER – Ionising Radiation (Medical Exposure) Regulations

**Table 4:** Future training requirements suggested by each NMP professional group

NMP Professional group	Course/training suggested
Nurses	Clinical decisions training
	Dealing with acutely unwell patients
	How to critically appraise research papers
	Interpreting blood results
	Pharmaceutical company training events
	Study days on developing the NMP role
Pharmacists	Diagnostic training module
	Filling out forms and relevant tasks training
	How to critically appraise research papers
	How to read CT scans
	Immunotherapy study day
	Interpreting blood results
	Keeping up to date with NICE & new treatment
	New trials related to specialised area
Radiographers	ChemoCare® electronic prescribing training
	Dealing with acutely unwell patients

**Box 2:** Comments from participants related to competency

**NMP appraisal**
*“… everyone needs that appraisal revalidation thing every year but signing a piece of paper? My experience of that is just a tick box thing, rather than just ‘what can I get from this?’”* (Pharmacist 2)
*“I have exactly the same appraisal with the Head of Nursing. I present my scope of practice and competencies and describe the competence of it …”* (Nurse 1)
**OSCEs**
*“… OSCEs are OK … but from a learning perspective they are actually not that useful once an NMP is experienced. The clinical judgement and clinical decisions that you make, and reasoning is more where competence should be assessed.”* (Nurse 1)
**Scope of practice**
*“… scope* [of practice], *I classically defined it and I wonder if that is really thought of in certain specialties.”* (Nurse 1)
**Peer review**
*“…* [Advanced Nurse Practitioners (ANPs)] *develop a portfolio and have that portfolio reviewed annually by a panel of experts within the trust which is done in a lot of other places for the ANPs … do it for independent prescribing …”* (Nurse 1)
**Auditing**
“*Prescribing is being aware of prescribing errors but there is good feedback from pharmacy… I keep those and self-audit.”* (Nurse 2)

**Box 3:** Comments from participants in relation to support

**Consultant support**
*“I feel well supported… but every aspect that my DMP* [Designated Medical Practitioner] *has supported me on is stuff that I have taken to them …, there needs to be a responsibility the other way from the consultant of what they want. More structure needed.”* (Pharmacist 3)
**Line manager support**
*“I asked to do the diagnostic course and …* [it]*never happened. If you don’t push for it then it doesn’t happen. My manager isn’t an NMP, so I wouldn’t expect any support from them … there is some support from the Chief Pharmacist …”* (Pharmacist 2)
*“* [my line manager who is a consultant radiographer] *if we have complex patients, we go to them and we discuss what we can do and what can be prescribed and then go and take it forward ourselves …”* (Radiographer 3)
**NMP peer support within the organisation**
*“… we have meetings every few months to catch up on NMP issues and arrange a little training … It could be better …*.” (Pharmacist 3)
*“… it is a case of sharing our experience. The newer ones are obviously probably gonna learn more, and in the discussion, you could share the benefit of your practice …”* (Nurse 2)
**Organisational support**
*“… We need more support on the realities on making an error and making decisions *etc*. NMPs don’t have the regulation in force to protect them …”* (Nurse 1)
*“… we should have protected time for E&T, but in reality, it wouldn’t happen, for example doctors have time built into their timetable … We should have dedicated time even if it is an hour or two a month …”* (Nurse 2)
**Professional group support**
*Internal to the organisation*
*“As a review team, we discuss difficult cases. We have our in-house competencies for reviewing patients and for prescribing …”* (Radiographer 2)
*External to the organisation*
*“BOPA is useful to keep up-to-date and being able to talk to people.”* (Pharmacist 1)
*“It is mostly a forum for radiographer NMPs. You can ask a question, and everyone answers …”* (Radiographer 1)

**Box 4:** Comments from participants in relation to training methods

**Learning from others**
*“Speaking to ANPs in different areas. That’s really important’”* (Nurse 1)
*“… pharmacists quite typically come with obviously far more knowledge of pharmacology and pharmacokinetics … The nurses come with a lot more hands-on patient assessment skill …”* (Nurse 3)
**In-house training**
*“Once you qualify there is no formal development unless you get a new role …”* (Nurse 2)
*“Regular training in-house for NMPs could be beneficial. Like once a month, but it could be difficult to establish topics to benefit everyone.”* (Radiographer 3)
**Self-directed learning**
*“It’s a grey area when you are extending your scope. Recently I was extending it to prescribe antibiotics but there was no formal training or support on this …”* (Nurse 2)
